# Novel Agents in Heavily Pretreated Metastatic Gastric Cancer: More Shadows Than Lights

**DOI:** 10.1155/2019/5692317

**Published:** 2019-07-04

**Authors:** Giandomenico Roviello, Alberto D'Angelo, Raheleh Roudi, Roberto Petrioli, Enrico Mini

**Affiliations:** ^1^Department of Health Sciences, University of Florence, Viale Pieraccini 6, 50139 Florence, Italy; ^2^Department of Biology and Biochemistry, University of Bath, Bath BA2 7AY, UK; ^3^Oncopathology Research Center, Iran University of Medical Sciences, Tehran, Iran; ^4^Medical Oncology Unit, Department of Medicine, Surgery and Neurosciences, University of Siena, Siena, Italy

## Abstract

Metastatic gastric cancer is still a disease with a poor prognosis. Recently, different novel agents (e.g., apatinib, nivolumab, TAS-102) have demonstrated a survival advantage compared with placebo for patients with heavily pretreated metastatic gastric cancer. Although the possible availability of active agents may be a desirable option in a very poor therapeutic scenario, clinical data from the recent studies with these drugs raise yet controversial issues. The purpose of this review is to briefly summarize the results of these novel drugs focusing on the limitations that bring some shadows on their positive therapeutic results.

## 1. Introduction

Increased survival of metastatic gastric cancer (GC) is still an unmet clinical need. Generally, the first line of treatment is represented by a combination of chemotherapy with platinum and fluoropyrimidine with or without a third drug [1]. After progression of disease from first line, taxanes (mainly paclitaxel) and irinotecan have been shown to be effective and tolerable [2], while ramucirumab, a human IgG1 monoclonal antibody against vascular endothelial growth factor receptor 2 (VEGFR2), alone or in combination with paclitaxel has statistically increased survival for GC patients [3, 4].

Unfortunately, no clear evidence has been established for patients who experience disease progression after a second line of therapy. In particular, no randomised, prospective clinical trial or guideline has supported the use of a specific drug. Recently, this scenario seems changed as novel agents have been shown to increase survival in third or in a further line of treatment. Based on the positive results of two randomized trials against placebo, apatinib, a small selective VEGFR2 tyrosine kinase inhibitor, has been the first to demonstrate a survival advantage in this GC patient population and from 2014 is approved in China [5]. In addition, nivolumab, a fully human IgG4 monoclonal antibody inhibitor of programmed death-1 (PD-1), also showed a survival benefit compared with placebo in patients with advanced gastric cancer refractory to two or more previous regimens of chemotherapy [6], and more recently, trifluridine/tipiracil, a novel oral combination cytotoxic drug also known as TAS-102, has significantly improved overall survival (OS) compared with placebo in heavily pretreated population of patients with advanced gastric cancer [7]. The purpose of this review is to briefly summarize the results of the studies with these novel drugs focusing on some limits that might at least in part reside in their clinical relevance.

### 1.1. Apatinib

Apatinib is a small-molecule receptor tyrosine kinase inhibitor that primarily binds to and inhibits VEGFR2 [5]. In addition, this agent inhibits c-Kit and c-SRC tyrosine kinases at higher concentrations [5]. In 2013, Li et al. conducted a phase II, randomized, double-blinded, and placebo-controlled trial to evaluate the efficacy and safety of daily apatinib administration as third-line treatment in patients affected by metastatic GC [8]. The study was conducted at 15 hospitals in China with a total of 141 patients enrolled and randomly assigned to receive placebo (group A, number=48), apatinib 850 mg once daily (group B, number=47), or apatinib 425 mg twice daily (group C, number=46). Apatinib demonstrated improved OS and progression-free survival (PFS) in heavily pretreated patients with metastatic GC who underwent two or more previous chemotherapy regimens' failure. The median OS was significantly higher in patients treated with apatinib versus those given a placebo with a median OS of 2.50 months for patients in group A, 4.83 months for patients in group B, and 4.27 months for patients in group C. Also, the PFS was significantly higher in patients administered with apatinib than in placebo with a median PFS of 1.40 months for group A, 3.20 months for group B, and 3.67 months for group C. As a noteworthy event, 9 patients (3 treated with apatinib 850mg and 6 treated with apatinib 425mg) had a partial response confirmed on computer tomography scan and 43% of patients given apatinib reached disease control.

In 2016, Li et al. performed a randomized, double-blind, placebo-controlled phase III trial to evaluate the efficacy and safety of apatinib in patients with advanced metastatic gastric cancer or gastroesophageal junction adenocarcinoma, refractory to two or more lines of previous chemotherapy regimens [9]. The study was undertaken in 32 centres in China and involved 267 patients randomly assigned at a 2:1 ratio to receive apatinib-matching placebo tablets once daily (number=91) or oral apatinib 850 mg in tablet form (number=176). This phase III study reported that apatinib, administered as monotherapy, prolonged OS and PFS: the OS was 6.5 months for the apatinib group and 4.7 months for the placebo group; likewise, the PFS improved from 1.8 months in the placebo group to 2.6 months in the apatinib group. In addition, the objective response rate (ORR) achieved was 2.84% with apatinib and 0% with placebo whereas the disease control rate (DCR) was critically higher in the apatinib group (42.05%) compared to the placebo group (8.79%). Unfortunately, no significant differences between the two groups with regard to the quality of life have been reported. In addition, dose reduction resulting from toxicity was more common in the apatinib group mainly due to grade 3 to 4 hand-foot skin reaction (8.5%), proteinuria (2,3%), and hypertension (4.5%).

In 2017, Ruan et al. carried out a multicenter, open-label, single-arm phase II study to evaluate the safety and efficacy of apatinib in patients affected by metastatic GC [10]. They enrolled a total of 42 patients from 4 different institutions in China with a histologically confirmed metastatic GC diagnosis, with no previous molecular target therapy but a second-line or last chemotherapy regimen failure. All patients were administered with apatinib 850 mg daily 30 minutes postprandially on days 1 to 28 of each 4-week cycle. They clearly demonstrated that apatinib is safe and with good efficacy in pretreated metastatic GC patients as the median PFS and OS reported were 4.0 months (95% CI, 2.8-5.1) and 4.5 months (95% CI, 4.0-4.9), respectively. The disease control rate and objective response rate were 78.6% and 9.5% after 2 cycles and 57.1% and 19.0% after 4 cycles. They also showed that toxicities were tolerable and clinically manageable with elevated aminotransferase (45.2%), hand-foot syndrome (40.5%), and secondary hypertension (35.7%) as main adverse events. Up to date, no planned study will investigate the role of Apatinib in non-Asian patients and in third or further line of treatment.

### 1.2. Nivolumab

Nivolumab is a fully human IgG4 monoclonal antibody known as a programmed cell death 1 (PD-1) immune checkpoint inhibitor. PD-1 is a transmembrane inhibitory immune-receptor, member of the B7-CD28 family, expressed on activated T, B, and natural killer cells [11].

Kang et al. assessed the safety and efficacy of nivolumab in 493 patients with advanced gastric or gastroesophageal junction cancer intolerant of, or refractory to, two or more previous regimens of chemotherapy in the randomised, placebo-controlled, double-blinded, phase III ATTRACTION-2 [6]. The study was performed at 49 hospitals in Taiwan, South Korea, and Japan and the 493 patients were randomly assigned at a ratio 2:1 to receive 3mg/kg of nivolumab (n=330) or placebo (n=163) intravenously every 2 weeks; patients who had previously been treated with anti-PD-L1 or anti-PD-L2, anti-PD-1, anti-CD137, or anti-CTLA4 were excluded from the trial. The primary endpoint of the study was OS. Patients in the nivolumab group had longer survival of 5.26 months compared to the placebo group with 4.14 months. The risk of disease progression was lower in nivolumab group (46%) than the placebo group (60%) and 11.2% of patients in the nivolumab group had an objective response (all confirmed partial responses) in comparison to a 0% in the placebo group. In addition, 29.1% of patients in the nivolumab arm achieved stable disease compared to the 25.2% of the placebo arm; thus the percentage of patients with disease control was 40% in the nivolumab group and 25% in the placebo group. Interestingly, two subgroup analyses indicated that nivolumab improved OS regardless of PD-L1 positivity and previous ramucirumab treatment. Although symptomatic adverse events were reported equally in both groups and the incidence of serious treatment-related adverse events was low (mainly pruritus, diarrhoea, rash, and fatigue), a limitation of this study was the absence of data about the quality of life. A subsequent analysis from the ATTRACTION-2 showed a similar OS improvement of Nivolumab compared to placebo in the Japanese subpopulation [12]. In addition, data from the gastric cohort from the multicenter, phase I/II CheckMate-032 [13] trial demonstrated a clinical activity of nivolumab and nivolumab plus ipilimumab in patients with chemotherapy-refractory esophagogastric cancer; however, phase III studies are awaited to confirm these data. Finally, although several studies are investigating the role of nivolumab in GC, no other studies are planned in third or further line of therapy.

### 1.3. Trifluridine/Tipiracil

TAS-102 is a novel oral cytotoxic chemotherapy consisting of a combination of trifluridine: thymidine-based nucleoside analogue and tipiracil: a thymidine phosphorylase inhibitor. For this reason, TAS-102 has a unique mechanism of action [14, 15].

In 2016, Bando et al. performed a multicenter, phase II, single arm, open-label study to assess the safety, pharmacokinetic, and efficacy profile of TAS-102 single therapy in patients affected by advanced GC [6]. Six Japanese institutions and a total of 29 patients were involved in this clinical trial. All patients were > 19 years old, affected by unresectable or recurrent oesophagogastric junction or gastric adenocarcinoma with one or two previous chemotherapy regimens containing platinum derivatives, irinotecan, taxanes or fluoropyrimidine, and a documented progression of disease; the ECOG performance status score was 0 to 2 organ functions reasonable. Patients received a 35 mg/m^2^ twice oral dose (b.i.d.) of TAS-102 per day after meals during a 28-d schedule with treatment on days 1-5 and 8-12. The 35 mg/m^2^ b.i.d. dose of TAS-102 showed efficacy with no unexpected toxicity in advanced GC patients and the primary point–the disease control rate–has been achieved and exceeded the primary endpoint target. The investigator determined disease control rate was 65.5% (95% confidence interval, 45.7-82.1%) whereas the independent central review's disease control rate was 51.9% (95% CI, 31.9-71.3%); the median PFS and OS were, respectively, 2.9 months (95% CI, 1.1-5.3 months) and 8.7 months (95% CI, 5,7.14.9 months). Neutropenia (69.0%), leucopenia (41.4%), anaemia (20.7%), and anorexia (10.3%) were the main grade III/IV adverse events reported.

In 2018, Shitara et al. [7] reported the results of a randomised, multinational, double-blinded, placebo-controlled, and phase III trial to assess the efficacy and safety of trifluridine/tipiracil at the dose of 35 mg/m^2^ twice daily on days 1–5 and days 8–12 every 28 days plus best supportive care as a new option treatment in patients heavily pretreated and affected by metastatic GC. OS was the primary endpoint. The trial was carried out in 110 academic hospitals of 17 countries, enrolling a total of 507 patients randomly assigned at a 2:1 ratio to the TAS-102 group (number=337) and the placebo group (number=170). A survival benefit was noted with TAS-102: the OS was 5.7 months in the TAS-102 group and 3.6 months in the placebo group; the disease progression at the data cut-off occurred in 85% of patients in the TAS-102 group and in 92% of patients in the placebo group; 14% of patients in the placebo group and 44% of patients in the TAS-102 group achieved an acceptable disease control. TAS-102 was well-tolerated even though a dosage modification due to an any-grade adverse event of any cause was reported in 22% of patients in the placebo group and in 58% in the trifluridine/tipiracil group. A potential limitation of the study was the scheduling of the first tumour assessment performed 8 weeks later after the randomisation, which might have precluded detection of radiological progression at earlier time points and the absence of data of patients pretreated with ramucirumab. Finally, quality of life data were investigated but were not reported as they will be in a separate paper. Interestingly, the NCT03686488 study is recruiting patients to evaluate the combination of TAS 102 and ramucirumab in patients with advanced, refractory gastric, or gastroesophageal junction adenocarcinoma and the phase I/II trial (NCT03368963) will investigate the combination of TAS-102 with nanoliposomal irinotecan in patients with gastrointestinal cancers (including GC).

## 2. Discussion

The prognosis of metastatic GC is still poor. To date, platinum plus a fluoropyrimidine is approved as first-line of therapy and second-line treatment with ramucirumab and taxane or irinotecan is widely used in patients who experience disease progression. The possibility of a third line of therapy is generally considered in patients with good performance status. Single-agent chemotherapy with docetaxel, paclitaxel, or irinotecan as well as different combinations was investigated in small phase II or retrospective studies in this GC patient population [16–20]; however, no therapy is recommended by internationally recognised treatment guidelines in GC patients who have failed two previous lines of treatment.

This scenario could however change as three novel agents with a different mechanism of action (apatinib, nivolumab, and TAS-102) have shown a survival advantage for the first time in recent randomized phase III trials which involved advanced GC patients progressed after at least two previous lines of therapy (Tables 1, 2, and 3 [6–8]). In particular, the TAGS and ATTRACTION-2 trials enrolled about 25% and 40% of patients treated with ≥4 previous chemotherapy regimens which compose a very heavily pretreated population of patients with metastatic GC [6, 7].

Although the possible availability of active agents may be a positive option in a very poor therapeutic scenario, the results reported with apatinib, nivolumab, and TAS-102 in the above studies raise some controversial issues.

First, all the three novel drugs have been tested against a placebo. Although this methodological choice of study type is theoretically supported by the absence of a specific treatment suggested by guidelines, a very recent phase III trial which compared avelumab (a human anti-PD-L1 monoclonal antibody) to the physician's choice chemotherapy did not show improvements in OS or PFS in third-line of therapy of GC patients [16]. Similarly, the KEYNOTE-061 trial showed that pembrolizumab (an anti PD-1) did not significantly improve OS compared with paclitaxel as second-line therapy [21]. All these data underline the importance of an active control for the evaluation of novel agents. On the other hand, other targeted agents (regorafenib and everolimus) tested in phase III trials against placebo in the third-line setting for this disease did not obtain any survival improvement [22, 23].

**Figure 1:**
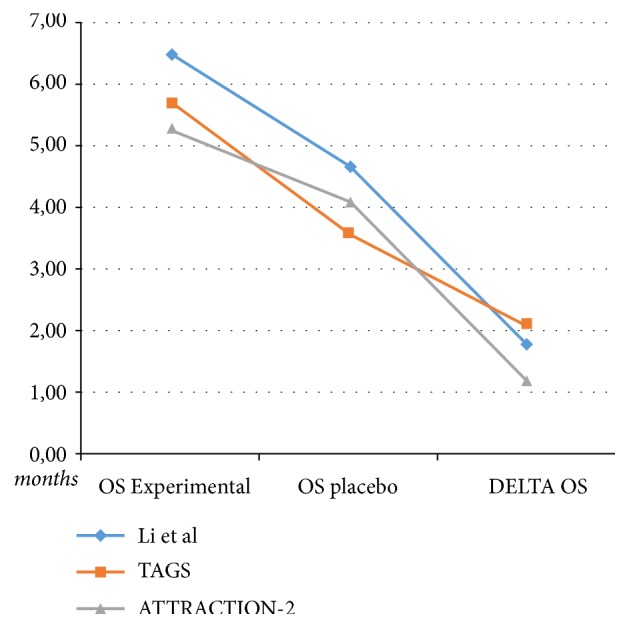
Data of median overall survival (OS) of the experimental arm and placebo arm and delta OS difference between OS of the experimental arm and placebo arm.

**Figure 2:**
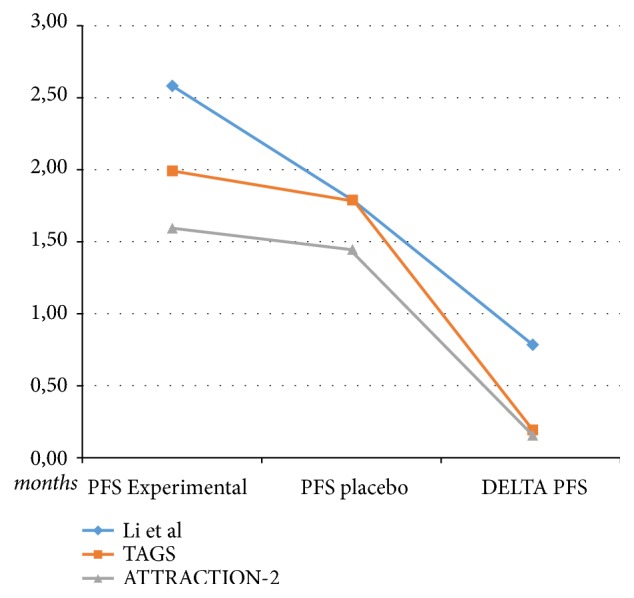
Data of median progression-free survival (PFS) of the experimental arm and placebo arm and delta PFS difference between PFS of the experimental arm and placebo arm.

A second limitation of the studies with apatinib, nivolumab, and TAS-102 in third or further line of treatment is related to the fact that the absolute survival gain (difference between OS of experimental arm with the OS of placebo) ranges from 1.2 to 2.1 months (Figure 1) and the absolute PFS gain ranges from 0.16 to 0.8 months only (Figure 2). These results suggest a limited clinical efficacy in terms of absolute survival advantages.

A third limitation of the apatinib and ATTRACTION-2 studies is related to the patients enrolled in these trials. The apatinib trial did not perform a double-blind randomization and enrolled Chinese patients only [8]. The ATTRACTION-2 (nivolumab) trial was conducted in Japan, South Korea, and Taiwan centres and excluded Western patients [6]. The TAGS was the only study that enrolled both Western and Asian patients [7]. Thus, differences in the ethnicity of enrolled patients should be considered in evaluating study results and their transferability to the Caucasian population.

A fourth limitation of the analysed studies is related to the poor information of data on quality of life: this outcome is widely incorporated in phase III studies involving patients with solid tumours and recently it has been demonstrated to be an important endpoint also in gastric cancer patients who have often symptoms related to the extension of disease [24]. While apatinib, nivolumab, and TAS-102 obtained a statistically significant increased tumour control rate as compared to placebo (Table 2), Li et al. did not show significant differences between apatinib or placebo at any time point about the quality of life score [8], the ATTRACTION-2 did not investigate the effects of nivolumab on the patient quality of life [6], and quality of life data have not yet been reported for the TAGS study [7], although the quality of life is very important in GC [24].

**Figure 3:**
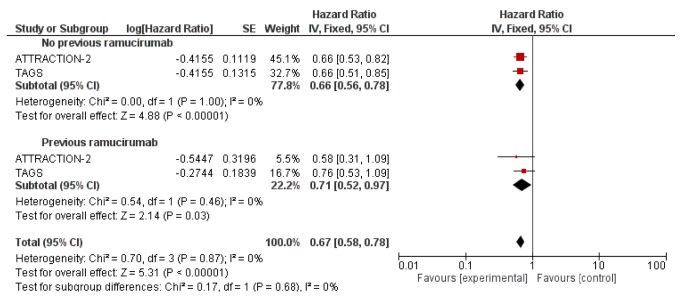
Pooled analysis according to previous ramucirumab.

Finally, another limitation when heavily pretreated patients are investigated raises from the percentage and type of previous lines of treatment. In particular, the rate of patients treated with a ramucirumab-based therapy may influence the outcome. Notably, the TAGS and the ATTRACTION-2 study enrolled patients who progressed on previous ramucirumab (about 30% in TAGS and 10% in ATTRACTION-2). A pooled analysis according to previous ramucirumab revealed that OS was significantly improved with a greater extent in patients not previously treated with ramucirumab (HR=0.66; 95%CI: 0.56-0.78;* p*<0.00001, I^2^: 0% Figure 3) compared with patients treated with ramucirumab (HR=0.71; 95%CI: 0.52-0.97;* p*=0.003; I^2^: 0% Figure 3). Therefore, previous ramucirumab-based therapy increases slightly the risk of death if compared with patients not previously treated with ramucirumab. Apatinib, nivolumab, and TAS-102 have been the first anticancer agents to gain a statistically significant advantage in survival in heavily pretreated cohort of patients with metastatic GC within phase III trials. Some concerns have however to be raised on study characteristics and results such as the absence of an active therapy control arm, differences in patient baseline characteristics, limited absolute advantage in survival results, and lack of patient quality of life information. These concerns have been reflected in the decisions taken by regulatory agencies worldwide on the approval of these drugs for this indication. Apatinib has been approved in China only and has obtained an orphan drug designation in Western countries. Nivolumab has been approved in Japan [25] but not in Western countries. TAS-102 has been recently approved for this indication in gastric cancer in the USA by FDA, and an application for the same indication has been filed to the European Medicine Agency (EMA).

As other neoplasms, GC is a heterogeneous disease in terms of clinical and molecular features, also as a function of its progression within time. In the near future, efforts in the development and clinical trials of antiangiogenic, immunoactivity, and classical cytotoxic cancer therapeutics should be pursued and specifically addressed to the identification of predictive biomarkers to improve the selection of ideal patient candidates in the various settings of the disease. This approach in the era of precision oncology represents the main route able to obtain more clinical benefits and real breakthrough advancements with all available therapies.

## Figures and Tables

**Table 1:** Characteristics of the analysed trials.

Study	Phase	Primary endpoint	Years/Range	Number of Patients Experimental Arm	Number of Placebo Arms	Line of treatment	Experimental arm
Li et al 2016	III	OS/PFS	2011-2012	176	91	III/IV°	Apatinib
TAGS 2018	III	OS	2016-2018	337	170	III/IV/V°°	TAS-102
ATTRACTION-2 2017	III	OS	2014-2016	330	163	III/IV/V°°°	Nivolumab

PFS: Progression free survival; OS: Overall survival.

°>III lines: 36% placebo; 34% apatinib

°°>III lines: 62% placebo; 63% TAS-102

°°°>III lines: 82% placebo; 80% nivolumab

**Table 2:** Data on overall survival, progression-free survival, and tumour response of the included studies.

Study	OS (months)		PFS (months)		Overall response rate (%)		Disease control rate (%)		Treatment duration of experimental drug (months)
	*Exp*	*C*	*Exp*	*C*	*Exp*	*C*	*Exp*	*C*	
	*arm*	*arm*	*arm*	*arm*	*arm*	*Arm*	*arm*	*Arm*	
Li et al 2016	6.5	4.7	2.6	1.8	1.7*∗*	0*∗*	31.8*∗*	11*∗*	2.9
TAGS 2018	5.7	3.6	2	1.8	4	2	44	14	3
ATTRACTION-2 2017	5.3	4.1	1.61	1.4	11	0	40.3	25	1.9

Exp: Experimental; C: control; NA: Not applicable

*∗*Assessed by independent response evaluation committee

**Table 3:** Characteristics of patients in the evaluated studies.

Study	Median age/Male patients %		ECOG > 0 %		Diffuse Histology %		Primary lesion GEJ junction %		Prior Surgery %		Number of metastatic sites >2 %		Peritoneal metastasis %		> II previous lines of treatment %		Previous Ram. %	
	*E*	*P*	*E*	*P*	*E*	*P*	*E*	*P*	*E*	*P*	*E*	*P*	*E*	*P*	*E*	*P*	*E*	*P*
Li et al 2016	58/75	58/75	73	83	NR	NR	22	23	69°	74°	21	22	24	27	34	36	NR	NR
TAGS	64/75	63/69	64	60	16	12	29	28	44°	44°	54	58	26	31	63	62	34	32
ATTRACTION-2	62/69	61/73	71	71	NR	NR	NR	NR	60°	64°	75°°	73°°	19	26	79	72	11	13

E: experimental arm; P: placebo arm; NR: Not reported; GEJ: Gastroesophageal; Ram: Ramucirumab

° Gastrectomy

°° ≥2 organs with metastases
